# Advances in nanotechnology for targeting cancer-associated fibroblasts: A review of multi-strategy drug delivery and preclinical insights

**DOI:** 10.1063/5.0244706

**Published:** 2025-03-13

**Authors:** Zhongsong Zhang, Yujie Tang, Dan Luo, Jing Qiu, Long Chen

**Affiliations:** 1 School of Basic Medical Sciences, Chengdu Medical College, Chengdu 610550, China; 2 School of Clinical Medicine, Chengdu Medical College, Chengdu 610550, China; 3 Department of Respiratory and Critical Care Medicine, First Affiliated Hospital of Chengdu Medical College, No. 278, Baoguang Avenue, Xindu District, Chengdu 610500, Sichuan, China

## Abstract

Cancer-associated fibroblasts (CAFs) play a crucial role in the tumor microenvironment by promoting tumor growth, immune evasion, and metastasis. Recently, drug delivery systems targeting CAFs have emerged as a promising long-term and effective approach to cancer treatment. Advances in nanotechnology, in particular, have led to the development of nanomedicine delivery systems designed specifically to target CAFs, offering new possibilities for precise and personalized cancer therapies. This article reviews recent progress in drug delivery using nanocarriers that target CAFs. Additionally, we explore the potential of combining multiple therapies, such as chemotherapy and immunotherapy, with nanocarriers to enhance efficacy and overcome drug resistance. Although many preclinical studies show promise, the clinical application of nanomedicine still faces considerable challenges, especially in terms of drug penetration and large-scale production. Therefore, this review aims to provide a fresh perspective on CAF-targeted drug delivery systems and highlight potential future research directions and clinical applications.

## I. INTRODUCTION

Cancer-associated fibroblasts (CAFs) are a key part of the tumor microenvironment (TME) and play a crucial role in the initiation, growth, and treatment of tumors.^1^ These cells help tumor cells grow, invade, and spread by releasing substances such as cytokines, matrix metalloproteinases (MMPs), and extracellular vesicles.^2,3^ They also change the extracellular matrix (ECM), which strengthens the tumor's barrier and limits the ability of standard anti-tumor drugs to penetrate.^4,5^ Additionally, CAFs can suppress the immune response against tumors and help tumors escape from the immune system.^6^ Because of their multiple roles in the TME, targeting CAFs has become an important focus in cancer treatment research.^7,8^ To solve this problem, drug delivery systems targeting CAFs have been developed. These systems create targeted strategies based on the unique behavior of CAFs. For example, they use surface markers like α-SMA and FAP or regulate specific biological signals in the tumor microenvironment to directly target CAFs.^9–11^ This approach not only suppresses the “support” effect of CAFs on tumors but also indirectly weakens the growth capacity of the tumor itself. So far, nanomedicine delivery systems (NDDS) have shown significant advantages in targeting CAFs.^12^ Nanodrugs improve drug accumulation in tumor tissues through enhanced permeability and retention (EPR) effects, thanks to their unique properties,^13,14^ such as controllable particle size, diverse surface modifications, and excellent biocompatibility.^15^ At the same time, nanocarriers can carry multiple drugs, enhancing treatment effectiveness through combined therapeutic strategies. For example, they can deliver both chemotherapeutic drugs and immunomodulators together to overcome tumor drug resistance.^16,17^ In addition, responsive nanocarriers can also intelligently release drugs under specific stimuli in the tumor microenvironment, such as acidity, redox states, or enzymes, further improving the precision of treatment.^18,19^ At present, significant progress has been made in nanomedicine targeting CAFs, showing great potential for future use.^12,20^ Various types of nanocarriers, including liposomes, polymer nanoparticles and metal nanomaterials, are widely used in targeted design.^21^ These carriers bind efficiently to CAFs by using specific targeting molecules on their surface, improving selectivity and therapeutic effectiveness.^22,23^ The development of multifunctional nanoplatforms has opened up new possibilities for treating tumors, allowing for the combined delivery of drugs and enabling imaging and real-time monitoring of treatment.^24,25^ However, nanomedicine targeting CAFs still faces challenges related to permeability, manufacturing processes, and long-term safety, requiring further breakthroughs for clinical application.^26,27^ We hope that more research results will soon be applied in clinical practice.

## II. THE BIOLOGICAL BASIS OF CAFs FOR TARGETED THERAPY

### A. CAF subtypes: Diversity, mechanisms, and therapeutic potential in cancer

**FIG. 1.**
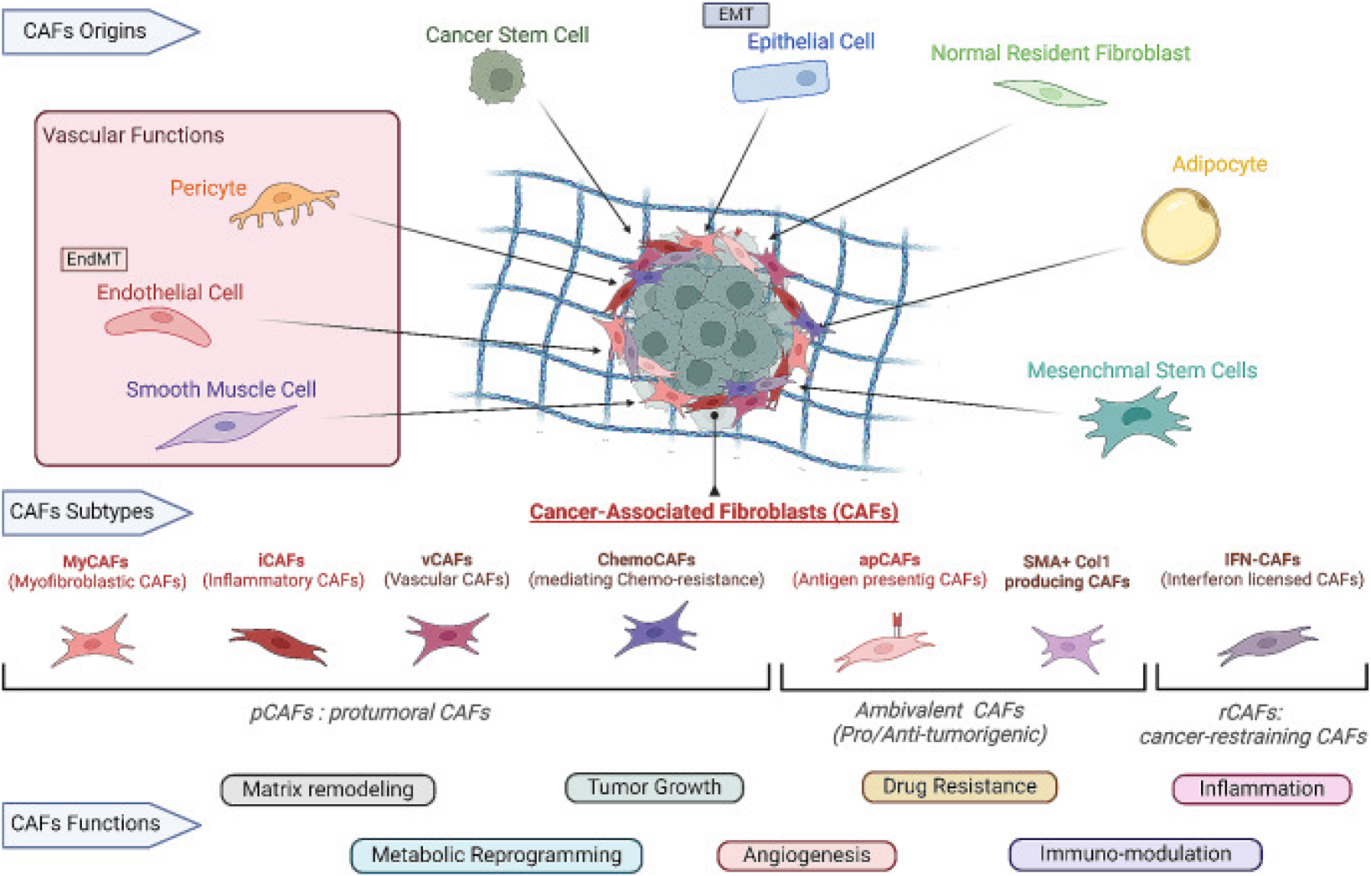
The origin, subtypes, and functions of CAFs in the TME. CAFs can originate from various cellular sources, including normal resident fibroblasts, adipocytes, mesenchymal stem cells, epithelial cells undergoing epithelial-mesenchymal transition (EMT), and cancer stem cells. CAFs can also arise from vascular origins, such as pericytes, endothelial cells, and smooth muscle cells, and through endothelial-to-mesenchymal transition (EndMT). The subtypes of CAFs are classified based on their distinct functional roles in tumor progression, which include: myofibroblastic CAFs (MyCAFs), which contribute to matrix remodeling and tumor growth; inflammatory CAFs (iCAFs), involved in inflammatory responses within the tumor microenvironment; vascular CAFs (vCAFs), which support angiogenesis and vascular development; chemo-resistant CAFs (ChemoCAFs), which mediate chemoresistance in tumors; antigen-presenting CAFs (apCAFs), which are involved in immune modulation by presenting antigens; SMA+ Collagen I-producing CAFs, which play a role in fibrosis and extracellular matrix production; and interferon-licensed CAFs (IFN-CAFs), which regulate immune responses and inflammation. These CAF subtypes fulfill various functions including metabolic reprogramming, matrix remodeling, tumor growth promotion, angiogenesis, drug resistance, inflammation, and immune modulation. Reproduced with permission from Coursier and Calvo, Cell Oncol. **47**(4), 1091–1112 (2024). Copyright 2024 by the authors.^61^

Over the past few years, an increasing number of studies have revealed the heterogeneity of CAFs, suggesting that CAFs are not a single-cell population, but are composed of multiple subtypes, each of which plays a different role in different stages and types of tumors.^28–30^ Common subtypes of CAFs include myofibroblast-type CAFs (myCAFs), inflammatory-type CAFs (iCAFs), antigen-presenting-type CAFs (apCAFs), and vascular-associated-type CAFs (vCAFs).^31,32^ Each subtype has unique molecular markers, functional characteristics, and potential implications for cancer treatment^32^ (Fig. 1). MyCAFs are primarily involved in the synthesis and deposition of ECM components,^33^ such as collagen and fibronectin. This process leads to tumor tissues exhibiting distinct fibrotic features, which, in turn, affect intercellular signaling and substance exchange, thereby creating a microenvironment conducive to tumor cell growth.^34,35^ Additionally, myCAFs can activate migration-related signaling pathways in tumor cells, such as PI3K-Akt and MAPK, through the secretion of growth factors, cytokines, and other signaling molecules.^36,37^ This activation promotes the migration and invasiveness of tumor cells.^38^

In contrast, iCAFs secrete various inflammatory factors, including interleukin-6 (IL-6), IL-11, CXCL1, CXCL2, and others.^39,40^ These factors activate cancer cells and contribute to immune regulation, thereby fostering a tumor microenvironment that promotes tumor cell growth and immune escape.^31,41^ For instance, IL-6 secreted by iCAFs activates the STAT3 signaling pathway, which promotes the migration and invasion of Tregs.^42^ This signaling pathway enhances the proliferation and immunosuppressive function of Tregs, while inhibiting the activity of CTLs, ultimately weakening the body's anti-tumor immune response.^43,44^

However, a distinctive feature of apCAFs is the expression of MHC class II molecules, granting them the potential ability to present antigens. This allows apCAFs to uptake, process, and present tumor-associated antigens, thereby participating in the regulation of tumor immune responses.^45,46^ Research has also shown that apCAFs can partially activate CD4+ T cells *in vitro* in an antigen-dependent manner.^47,48^ However, due to their low expression of co-stimulatory molecules (e.g., CD80, CD86, and CD40), apCAFs may not fully activate T cells. This limitation results in a reduced strength and persistence of the T cell immune response.^47,49^ vCAFs are primarily involved in promoting blood vessel formation and remodeling in tumors. They are often located around tumor blood vessels and play a key role in angiogenesis by secreting growth factors such as VEGF, FGF, and other molecules that promote blood vessel formation.^50,51^ vCAFs not only enhance tumor growth by supplying nutrients and oxygen through improved blood flow, but they also help create a tumor microenvironment that facilitates metastasis and immune evasion.^52,53^ Targeting vCAFs has become a promising therapeutic strategy for inhibiting tumor vascularization and limiting tumor progression.^54^

Indeed, the proportions of each subtype of CAFs vary in different tumor types, and several studies have suggested that balancing the number of CAF subgroups may provide important clinical benefits.^55–57^ For instance, vCAFs are found to be particularly abundant in tumors like lung cancer and gastric cancer, where they contribute to tumor blood supply and metastasis.^58^ On the other hand, myCAFs are more prevalent in fibrotic tumors such as pancreatic cancer.^59^ Despite the progress made in CAF subtype research, many questions remain unresolved. Future studies must further investigate the mechanisms underlying the interconversion between CAF subtypes and clarify the specific differences in their roles across various tumor types and individuals. This will provide a crucial theoretical foundation for early tumor diagnosis, personalized treatment, and prognostic assessment.^55,60^

### B. CAF markers: Crucial indicators in tumor development and treatment strategies

An important direction in cancer therapy research is the targeted treatment of tumors using specific CAF markers.^62,63^ CAF markers are not only markers of cell types in the tumor microenvironment, but they also reveal the biological functions of these cells in tumor initiation and development.^62,63^ The use of these biomarkers to target CAF can help inhibit tumor growth and metastasis and improve the efficacy of existing treatments.^64^

Alpha-smooth muscle actin (α-SMA), fibroblast activation protein (FAP), and other markers are commonly used to identify CAFs. α-SMA is a protein highly expressed in smooth muscle cells and myofibroblasts, and its expression reflects the activation status of CAFs. Therefore, α-SMA expression is significantly upregulated in CAFs and is one of the most widely used markers for identifying them.^65^ During tumorigenesis, normal fibroblasts are stimulated by signals such as transforming growth factor-β (TGF-β) secreted by tumor cells, undergoing phenotypic transformation and beginning to express α-SMA. This change enables them to contract and secrete extracellular matrix, thereby promoting tumor growth and metastasis.^66,67^ Additionally, the expression level of α-SMA is closely related to tumor malignancy and prognosis. Studies have shown that breast cancer patients with high α-SMA expression tend to have a higher risk of recurrence and lower survival rates.^68,69^ FAP, a membrane-bound serine protease, is highly expressed in CAFs, while it is either expressed at low levels or absent in normal fibroblasts. FAP plays a significant role in remodeling the extracellular matrix, as well as in the proliferation and migration of tumor cells, thus promoting tumor growth and metastasis.^50,68^ For instance, in pancreatic cancer, FAP-expressing CAFs can degrade extracellular matrix components such as collagen, opening a pathway for tumor cell metastasis.^70,71^ Platelet-derived growth factor receptor (PDGFR), including its two isoforms, PDGFRα and PDGFRβ, is another classic CAF marker. When bound to platelet-derived growth factor (PDGF), PDGFR activates downstream signaling pathways that promote the proliferation, migration, and activation of CAFs.^72,73^ In lung cancer, for example, the activation of PDGFR induces CAFs to secrete various cytokines and growth factors, such as TGF-β and VEGF, which further enhance tumor growth and angiogenesis.^74,75^

In addition to these classic markers, several emerging markers of CAFs have been identified. For example, in gastric cancer tissues, Vimentin-positive CAFs are closely associated with the depth of tumor invasion and lymph node metastasis. Vimentin expression levels can also serve as a prognostic indicator for tumors.^76^ In ovarian cancer, NRP-1-positive CAFs promote tumor growth and metastasis through interactions with tumor cells.^77,78^ In breast cancer, POSTN-positive CAFs secrete various growth factors and cytokines, activating signaling pathways within tumor cells and promoting their proliferation and migration.^79,80^ Interestingly, the role of Podoplanin-expressing CAFs varies across different tumor types. In small-cell carcinoma of the lung, Podoplanin-positive CAFs may inhibit tumor cell growth by secreting inhibitory cytokines or modulating immune cell activity.^81^ However, in lung adenocarcinoma and squamous cell carcinoma, Podoplanin-positive CAFs are associated with poor prognosis and may promote tumor invasion and metastasis.^82,83^

With advances in detection technologies, a range of CAF markers have been identified using methods such as single-cell sequencing,^30^ including laminin (LN), matrix metalloproteinases MMP, epithelial cell adhesion molecules (EPCAM), and mannose-binding lectin (MBL).^84–87^ Furthermore, in breast CAFs, specific genes such as NOTCH3 and HES4 have been identified as markers associated with CAF self-renewal and proliferation.^78^ This discovery suggests a potential new approach to combine gene technology with these specific markers and provides a promising direction for future research. Although various CAF markers have been identified, significant challenges remain in their application for cancer treatment. The heterogeneity of CAFs results in variations in marker expression across different tumor types and patients, complicating the uniform identification and application of these markers.^28,88^ Furthermore, some CAF markers are not exclusive to CAFs and may also be expressed in other cell types, which increases the complexity and the risk of false positives in marker detection. Future research should focus on further investigating the heterogeneity of CAFs and identifying more specific CAF markers. This will enhance the accuracy and reliability of CAF identification, thereby contributing to the development of targeted therapeutic strategies and improving the effectiveness of cancer treatments.

### C. CAFs: Central mediators of tumor microenvironment dynamics and progression

The tumor microenvironment (TME) is a complex network comprising various cells and molecules, including tumor cells, immune cells, endothelial cells, fibroblasts, and extracellular matrix components. Within this microenvironment, CAFs serve as key regulators, contributing to tumor initiation and progression. They influence tumor growth, metastasis, immune escape, and treatment response through interactions with other elements of the TME.^62,89^ As such, understanding the mechanisms by which CAFs function in the TME and targeting these pathways for cancer treatment has become a critical focus of current cancer research.^27,90^

Indeed, the interactions between CAFs and the TME are reciprocal. CAFs regulate the TME by secreting various growth factors, chemokines, and other molecules, all of which are essential for tumor formation and progression.^91,92^ For example, CAFs secrete signaling molecules involved in ECM remodeling, which in turn strengthens the structural framework required for tumor expansion. This remodeling provides a conducive environment for tumor cells to migrate and proliferate.^5,93,94^ Additionally, CAFs support tumor cells by participating in metabolic processes, such as regulating glucose, pH levels, and mitochondrial function. They also generate mechanical forces that influence tumor cell motility and morphology, thereby promoting tumor dissemination.^95^ Simultaneously, tumors and tumor cells can influence CAFs activation and function.^96,97^ CAFs are activated through pathways like direct contact or Notch signaling, which leads to ECM remodeling and creates a positive feedback loop.^98,99^ In pancreatic ductal adenocarcinoma (PDAC), for instance, tumor cells induce CAFs and autophagy to produce an “inverse Warburg effect,” releasing amino acids like alanine to support cancer cell metabolism.^100–102^ Furthermore, CAFs engage in transmembrane signaling with other cells within the TME. Through pathways involving PDGF, IL-6, and tumor necrosis factor-alpha (TNF-α), CAFs communicate with neighboring cells, thus influencing disease progression.^98,103^ These chemokines act as signals that recruit immune cells and other cell types to the tumor site. However, rather than enhancing an effective anti-tumor response, these recruited immune cells often contribute to an immunosuppressive environment. This occurs because CAFs can modulate immune cell function, transforming them from potential tumor fighters into supporters of tumor growth and metastasis.^97,104^

In addition to the influence of CAFs on the TME, the TME itself significantly impacts the biological characteristics and functions of CAFs. For example, high concentrations of hyaluronic acid (HA) or oxidative stress in the TME can stimulate CAFs to exhibit more aggressive, tumor-promoting behavior.^105^ These environmental factors alter CAFs' molecular activity, enhancing their secretion of growth factors, chemokines, and other molecules that support tumor growth and metastasis.^104,106^ Despite the progress in understanding these interactions, many uncertainties remain regarding how different TMEs influence CAF behavior.^27,107^ Future research is expected to provide deeper insights and breakthroughs in understanding these complex interactions, which could potentially lead to the development of novel therapeutic strategies targeting CAFs and their interactions within the TME.^108^

## III. PROGRESS IN NANOMATERIAL-BASED TARGETING OF CAFs

### A. Nanomaterials and their applications in CAF targeting

In recent years, nanomaterials have become an important tool for targeting CAFs due to their unique physicochemical properties.^109,110^ These materials provide precise drug delivery, better drug targeting, improved tissue penetration, and more control over drug release, all of which contribute to enhanced therapeutic effectiveness.^5,12,111,112^ The use of various nanomaterials in targeted therapy not only showcases their individual benefits but also opens up many opportunities for further research and clinical applications.^112,113^ Below, we discuss several types of nanomaterials, their applications, and their effects in CAF-targeted therapy (Table I).

**TABLE I.** Nanomaterials and their applications in targeting CAFs.

Material name	Cancer	Mechanism	Effect	Refs.
Gold nanoparticles (AuNPs)	Pancreatic cancer, breast cancer	AuNPs target CAF-specific proteins (e.g., FAP) through peptide modification and use near-infrared photothermal effects to ablate CAFs and reduce extracellular matrix density.	Reduces CAF activity, improves drug penetration, enhances anti-tumor immune responses.	26, 119, 145
Metal-organic frameworks (MOFs)	Pancreatic cancer, breast cancer	MOFs encapsulate chemotherapeutic drugs and are modified with CAF-targeting peptides. Near-infrared light triggers drug release, inducing CAF apoptosis.	Remodels the tumor microenvironment, reduces CAF density, and enhances drug efficacy.	146–148
Selenium nanoparticles (SeNPs)	Lung cancer	SeNPs inhibit CAF secretion of pro-inflammatory factors and enhance antioxidant capabilities, reducing CAF tumor-promoting activity.	Suppresses tumor-associated inflammation and blocks CAF support for tumor growth.	149, 150
Peptide-functionalized silica nanoparticles	Pancreatic cancer, prostate cancer	Targets FAP-α on CAF surfaces, releasing quercetin-loaded nanoparticles to inhibit CAF activity	Decreases extracellular matrix density and improves tumor drug penetration.	141, 151
Polymeric nanoparticles (PLGA-NPs)	Breast cancer, colorectal cancer	PLGA nanoparticles loaded with resveratrol target active CAF regions, reducing their immunosuppressive effects.	Remodels the immune microenvironment and enhances immunotherapy efficacy.	152, 153
Magnetic nanoparticles (MNPs)	Breast cancer, Colorectal cancer	MNPs are guided by external magnetic fields to deliver paclitaxel or small-molecule inhibitors, targeting CAF functions.	Enhances drug delivery efficiency and reduces CAF-mediated drug resistance.	154, 155
Liposome nanoparticles	Pancreatic cancer, breast cancer	FAP-targeted liposomes deliver chemotherapeutic drugs (e.g., paclitaxel) to inhibit CAF secretion of tumor-promoting factors.	Reduces tumor stroma barriers and enhances the effectiveness of chemotherapeutic drugs.	151, 156–158
Iron oxide nanoparticles (FeNPs)	Lung cancer, breast cancer	Targets CAFs via the TGF-β signaling pathway, releasing anti-fibrotic drugs to suppress CAF activity.	Reduces CAF density and extracellular matrix, improving immune cell infiltration.	159, 160

Gold nanoparticles (AuNPs) have attracted considerable attention in nanomedicine due to their excellent physicochemical properties, such as a large surface area, ease of functionalization, and high biocompatibility.^114,115^ These properties make AuNPs ideal candidates for targeted drug delivery, especially in therapies aimed at CAFs within the tumor microenvironment.^116,117^ Some studies have shown that AuNPs can be functionalized with ligands like peptides or antibodies,^118,119^ enabling them to specifically target CAF markers such as FAP and α-SMA.^120,121^ Additionally, AuNPs can carry a wide variety of therapeutic agents, from chemotherapy drugs to RNA molecules, making them a versatile platform for drug delivery.^111,119^ One promising use of AuNPs in CAF-targeted therapy is their combination with photothermal therapy.^122^ When exposed to near-infrared (NIR) light, AuNPs produce localized heat, which causes thermal ablation of CAFs and surrounding tumor cells, reducing tumor growth.^123,124^ This photothermal effect can be combined with chemotherapeutic drug delivery, improving local therapeutic effects while minimizing systemic toxicity. For example, Tan *et al.* demonstrated that functionalized AuNPs loaded with anticancer agents successfully targeted and eliminated CAFs within the TME.^125^ The ability to combine multiple treatment strategies, such as drug delivery and photothermal therapy, makes AuNPs a promising tool for CAF-targeted combination therapies.^126,127^ Furthermore, AuNPs are used in imaging applications, such as diagnostic imaging and real-time monitoring of treatment progress.^128,129^ The surface plasmon resonance of AuNPs allows for strong scattering and absorption of light, making them ideal for noninvasive imaging techniques that enhance the precision of treatment delivery.^130^

Photosensitizers, such as zinc phthalocyanine and heme, generate reactive oxygen species (ROS) when exposed to light, which can selectively kill cancer cells and CAFs.^131,132^ Photodynamic therapy (PDT), which uses these photosensitizers, has become an effective treatment for cancer due to its ability to target both tumor cells and CAFs.^133^ Zinc phthalocyanine (ZnPc), for example, has been combined with various nanomaterials, such as liposomes and polymeric nanoparticles, to improve its therapeutic effects while reducing side effects.^134,135^ In a study by Zhou *et al.*, ZnPc-loaded nanoparticles were designed to specifically target CAFs.^136^ Upon light exposure, ZnPc generates ROS that specifically damage CAFs, disrupt the tumor stroma, and improve the delivery of chemotherapeutic drugs to tumor cells. This combination of PDT with CAF-targeting strategies shows great promise for overcoming resistance in the tumor stroma and improving the effectiveness of conventional chemotherapy. Similarly, heme-based photosensitizers, incorporated into nanocarriers, have shown potential for CAF-targeted PDT. When irradiated, these nanomaterials generate ROS to promote localized tumor destruction.^137^ The targeting ability of these nanocarriers can be further improved by functionalizing them with CAF-specific ligands, providing a dual therapeutic approach that not only eliminates CAFs but also enhances the penetration of other therapeutic agents into the tumor.^138,139^

Nanoparticles modified with proteins or peptides offer an advanced approach for targeting CAFs in cancer therapy.^140^ By functionalizing nanoparticles with specific proteins or peptides, their targeting ability for CAFs can be significantly improved.^141^ Peptides like the RGD peptide, which binds to integrin receptors on CAFs, can be attached to nanoparticles to increase their affinity for CAFs.^142,143^ This peptide-based targeting has been widely studied because of the high specificity and low immunogenicity of peptides. Moreover, protein-based targeting strategies, such as using antibodies that target FAP, can also be used to effectively target and internalize nanoparticles by CAFs.^144^ Once internalized, these nanoparticles can deliver drugs directly to CAFs, disrupting their function and enhancing anti-tumor immunity. Therefore, the combination of peptides and proteins with nanoparticles can greatly enhance the precision of drug delivery, ensuring that therapeutic agents are targeted specifically to CAFs while minimizing off-target effects.

### B. Nanomaterials targeting different subtypes of CAFs

CAFs exhibit considerable heterogeneity within the TME and can be categorized into distinct subtypes: myCAFs, iCAFs, apCAFs, and vCAFs.^161–163^ Each subtype has a distinct role in tumor growth, immune evasion, and treatment resistance.^30,164^ Recent studies have shown that nanomaterials can effectively modulate the pro-tumor functions of these CAF subtypes by targeting specific biomarkers or related signaling pathways.^12,160^ Therefore, we explored targeting mechanisms for each CAF subtype and highlight the latest advances in nanomaterial-based therapies.

MyCAFs play a key role in ECM remodeling and fibrosis, which forms physical barriers that limit drug penetration.^165^ So targeting myCAFs is an important method to enhance drug delivery. For example, gold nanoparticles and graphene oxide nanocomposites have been shown to disrupt the ECM by targeting the expression of fibronectin and α-SMA in myCAFs.^123,166^ Cheng *et al.*'s study showed that photoactivated gold nanorods generate localized heat to degrade ECM components, thereby improving drug delivery efficiency and enhancing immune cell infiltration into the tumor core.^167^ iCAFs contribute to tumor inflammation and immune escape by secreting cytokines and chemokines such as IL-6 and CXCL12.^31^ Lipid nanoparticles loaded with IL-6 receptor (IL-6R) siRNA can effectively silence iCAF-related signaling pathways, leading to reduced tumor growth and metastasis.^168^ Additionally, magnetic nanocarriers combined with CXCR4 antagonists can inhibit tumor immune interactions mediated by CXCL12, thereby blocking iCAF-induced immune evasion.^169,170^ ApCAFs express major histocompatibility complex (MHC) class II molecules and play a role in antigen presentation.^30,47^ However, their immune regulatory functions are still being studied.^171^ Recent research has developed nanostructures based on DNA origami technology for delivering antigens and immune modulators to apCAFs, reprogramming them to stimulate immune responses.^172,173^ This innovative strategy opens up new possibilities for advancing tumor immunotherapy.

vCAFs promote angiogenesis by secreting vascular endothelial growth factor (VEGF) and fibroblast growth factor (FGF) and regulate tumor blood vessel formation through integrin signaling (e.g., α_v_β_3_).^53,174^ These processes supply the tumor with nutrients, supporting its growth. Nanomaterial designs targeting vCAFs aim to inhibit their pro-angiogenic functions. For example, polymer nanocarriers loaded with bevacizumab, a VEGF inhibitor, can neutralize VEGF signaling and significantly inhibit angiogenesis.^175,176^ Moreover, integrin-targeted nanoparticles can bind to α_v_β_3_ integrin, directly interfering with the vascular formation pathways mediated by vCAFs.^177^ Furthermore, iron oxide nanoparticles generate ROS that impair vCAF function, further reducing tumor angiogenesis.^178^ These multifaceted nanomaterials provide promising therapeutic strategies for inhibiting vCAF-induced angiogenesis, offering broad potential for anti-tumor angiogenesis applications.

### C. Targeted strategies for CAFs in different cancers

**FIG. 2.**
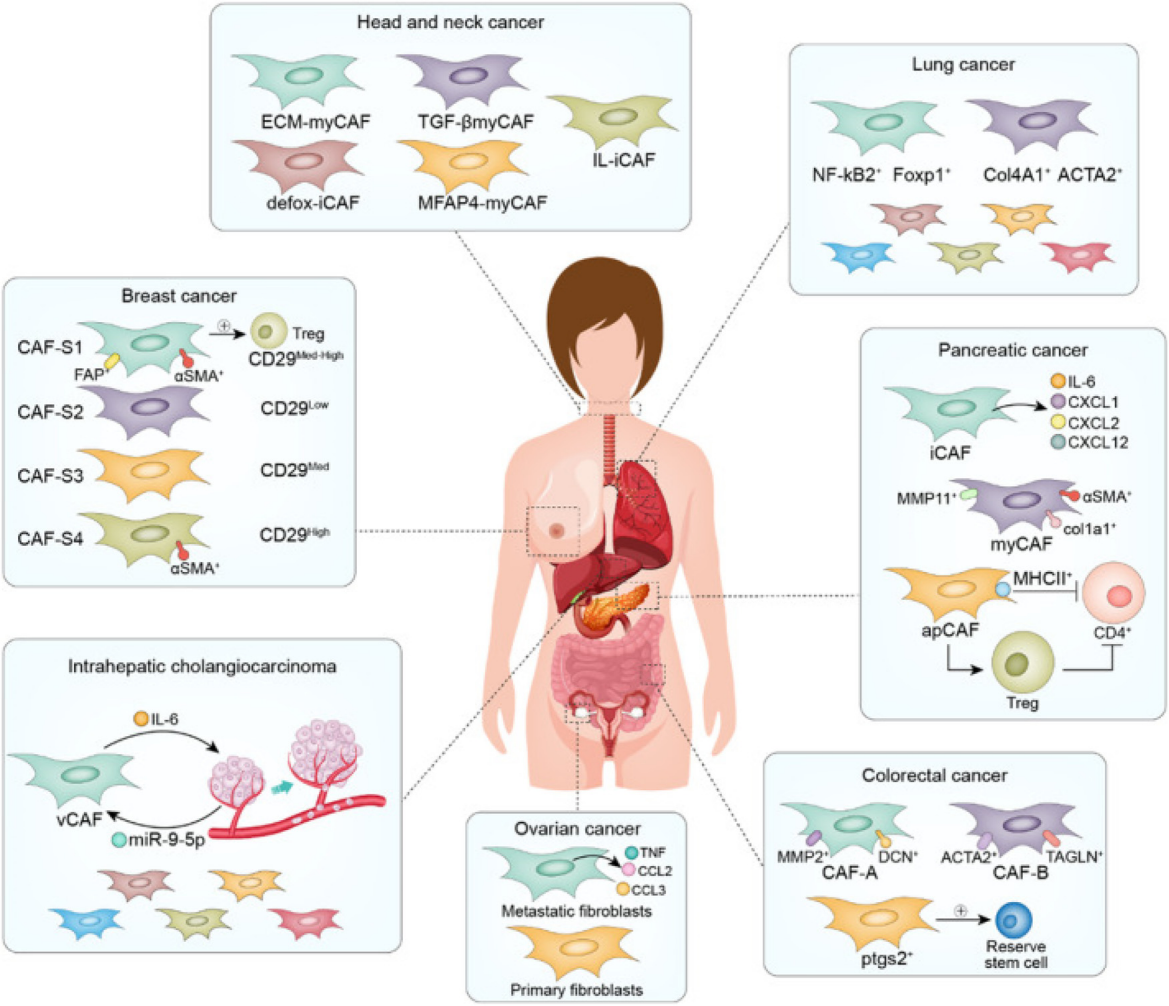
Heterogeneity of CAFs in different organs and their functional characteristics and potential targeting strategies in the TME. The figure highlights the diverse functions and characteristics of CAFs across various cancer types. These differences are crucial for determining specific therapeutic targets and designing nanomaterial-based strategies tailored to each cancer type. In head and neck cancer, CAFs are marked by ECM-myCAF, TGF-βmyCAF, iL-iCAF, and others, which play a role in tumor microenvironment modulation and immune response. In breast cancer, distinct CAF subtypes, such as CAF-S1 to CAF-S4, express various markers like FAP, αSMA, and CD29, impacting immune cell infiltration and tumor progression. Lung cancer CAFs express NF-κB, Foxp1, Col4A1, and ACTA2, which contribute to immune evasion and fibrosis. Pancreatic cancer shows CAFs with iCAF, myCAF, and apCAF and specific markers like MMP11, αSMA, and IL-6 involved in chemotherapy resistance and immune modulation. In ovarian cancer, both metastatic and primary fibroblasts are present, expressing TNF, CCL2, and CCL3, contributing to tumor progression and immune suppression. Colorectal cancer CAFs express MMP2, ACTA2, TAGLN, and ptgs2, potentially influencing cancer stem cells and tumor microenvironment remodeling. Intrahepatic cholangiocarcinoma CAFs, marked by vCAFs, express IL-6 and miR-9-5p, which are involved in tumor progression and fibrosis. Reproduced from Zhang, Xiangjian *et al.* Single-cell RNA sequencing to explore cancer-associated fibroblasts heterogeneity: “Single” vision for “heterogeneous” environment. Reproduced with permission from Zhang *et al.*, Cell Proliferation **57**(5), e13592 (2024). Copyright 2024 by the authors.^37^

The functions and characteristics of CAFs differ across various cancer types (Fig. 2), meaning that their therapeutic targets and nanomaterial design strategies must be tailored to each specific cancer type.^39,179^ So far, with advances in nanomaterial research, selective targeting of CAFs has become more achievable. Additionally, quantitative analysis of both *in vivo* and *ex vivo* accumulation has confirmed the effectiveness of these strategies, highlighting their significant potential in personalized cancer therapy (Table II).

**TABLE II.** Nanomaterials targeting CAFs in different cancers.

Cancer type	CAF role	Nanomaterial strategy	*In vivo*/*ex vivo* accumulation	Outcome	Refs.
Breast cancer	Promotes immune evasion and therapy resistance	FAP-targeting nanoparticles loaded with chemotherapy drugs	20× drug accumulation at tumor sites, significantly enhanced therapeutic effect	Selective killing of CAFs, improved drug delivery and tumor inhibition	181
PDAC (pancreatic ductal adenocarcinoma)	Leads to dense fibrotic stroma, dominated by myCAFs	Collagenase-loaded nanocarriers and pH-sensitive liposomes delivering TGF-β inhibitors	Collagen degradation efficiency >70% (*ex vivo*); 50% reduction in CAF activation (*in vivo*)	Improved drug penetration, reduced fibrosis, suppressed tumor growth	183,184
Colorectal cancer (CRC)	Secretes exosomes carrying miR-92a-3p, linked to metastasis and chemo-resistance	Exosome-mimicking nanoparticles delivering anti-miRNA molecules	15× nanoparticle accumulation in tumor tissues, reduced metastasis formation	Suppressed metastasis, enhanced chemotherapy sensitivity	188
Lung cancer	Overexpresses PDGFR, promoting tumor growth and immune suppression	PDGFR-targeting aptamers conjugated with drug-loaded liposomes	6× nanoparticle concentration at tumor sites, enhanced immune cell recruitment	Inhibited CAF activation, boosted immune cell infiltration	190
Gastric cancer	Secretes miR-522 via exosomes, suppressing ferroptosis and inducing drug resistance	Iron oxide nanoparticles restoring ferroptosis; dual-drug-loaded liposomes	12× nanoparticle accumulation in tumor tissues, 30% reduction in cancer cell survival rate	Restored ferroptosis, reduced tumor volume, extended survival	192

Taking breast cancer as an example, CAFs play a significant role in tumor progression by promoting immune escape and contributing to drug resistance.^180^ A study found that nanoparticles conjugated with a fibroblast activation protein (FAP)-specific ligand resulted in drug accumulation at the tumor site that was 20 times higher than in normal tissues in an *in vivo* breast cancer model, significantly enhancing the therapeutic effect.^181^ Specifically, FAP-targeting nanodots not only selectively kill CAFs but also minimize damage to healthy tissues, improve drug delivery efficiency, and inhibit tumor growth. In PDAC, the tumor's extremely dense fibrotic stroma, primarily dominated by myCAFs, makes ECM remodeling essential.^182^ Research has shown that nanocarriers loaded with collagenase can degrade over 70% of collagen in an *in vitro* PDAC model, greatly improving the permeability of chemotherapy drugs.^183^ Additionally, the use of pH-sensitive liposomes to deliver TGF-β inhibitors significantly reduces CAF activity in mouse models (by more than 50%) and effectively inhibits tumor fibrosis.^184^

In colorectal cancer (CRC), CAFs promote tumor metastasis and chemotherapy resistance by secreting exosomes that carry miR-92a-3p.^185,186^ To counter this, nanoparticles mimicking exosomes have been designed to deliver anti-miRNA molecules.^187^ *In vivo* experiments have shown that these nanoparticles accumulate in tumor tissue 15 times more than in normal tissues.^188^ This not only significantly reduces metastatic lesion formation but also increases chemotherapy sensitivity. For lung cancer, CAFs typically express high levels of PDGFR, providing a clear target for therapy.^189^ Drug-loaded liposomes combined with PDGFR aptamers show exceptionally high specificity in lung cancer models, with nanoparticle concentrations in the tumor site being six times higher than in other tissues.^190^ This strategy not only effectively inhibits CAF activation but also significantly enhances immune cell recruitment (such as T cells and NK cells) to the tumor. In gastric cancer, CAFs secrete miR-522 through exosomes, inhibiting ferroptosis of cancer cells and promoting drug resistance.^191^ To counteract this, iron oxide nanoparticles are used to restore ferroptosis, reducing the survival rate of tumor cells by more than 30%.^192^ Additionally, the design of dual-drug-loaded liposomes enables simultaneous targeting of both CAFs and tumor cells. In a mouse model of gastric cancer, this strategy significantly reduced tumor volume and prolonged survival time.^193^

Up to now, many research examples have demonstrated the advantages of nanomaterials in optimizing drug delivery and selectively targeting CAFs, and their effectiveness in improving treatment outcomes has been quantitatively analyzed through in various vivo and *in vitro* experiments. These findings lay a solid foundation for developing precise, personalized treatment plans and offer new hope for the future of cancer therapies.

### D. Biomarkers and mechanisms of nanomaterials targeting CAFs

CAFs exhibit significant heterogeneity and express various specific biomarkers, which provide multiple potential targets for targeted therapy. In recent years, substantial progress has been made in the design of nanomaterials targeting these CAF markers, leading to a notable improvement in tumor treatment efficacy by precisely targeting these markers.^111^ These biomarkers not only offer a molecular understanding of CAF function but also serve as potential targets for therapeutic intervention.

For example, FAP, which is highly expressed in CAFs, plays a key role in degrading the ECM and promoting tumor cell invasion. Nanomaterials targeting FAP utilize FAP antibodies or small molecule ligands to achieve precise targeting. FAP-targeted nanoparticles can deliver chemotherapy drugs, such as paclitaxel and doxorubicin, selectively destroying CAFs while avoiding damage to normal tissues.^151,194^ Furthermore, FAP-targeted photothermal nanomaterials, such as gold nanoparticles, generate local thermal effects after photoactivation.^195^ This disrupts CAF function and significantly improves drug permeability in the tumor stroma.

The high expression of α-SMA in CAFs is closely related to tumor stroma rigidity and fibrosis, which enhance tumor resistance to drugs. To target this biomarker, liposome nanoparticles can carry TGF-β inhibitors, such as Galunisterib, to inhibit CAF activation and fibrosis.^196^ Platelet-derived growth factor receptor (PDGFR), which is highly expressed in CAFs, plays a critical role in CAF proliferation and angiogenesis. PDGFR-targeted polymer nanoparticles, modified with an adapter, significantly reduce tumor vascular density by delivering PDGF inhibitors or siRNA.^197^ VEGF, secreted by CAFs, is a major factor in tumor angiogenesis, providing essential nutritional support for tumors. Nanocarriers loaded with VEGF inhibitors, such as bevacizumab, can neutralize VEGF secreted by CAFs and significantly inhibit angiogenesis.^198^ Moreover, VEGF nucleic acid aptamer-modified nanoparticles can further disrupt the tumor's blood supply by precisely binding to VEGF.^199^

In immune escape, CXCL12 secreted by CAFs attracts immunosuppressive cells, such as Tregs and MDSCs, through the CXCR4 receptor, creating an immune escape environment.^200^ CXCR4 antagonist nanomaterials can block the CXCL12-CXCR4 axis, restore immune cell function, and improve anti-tumor immune responses.^201^ In a melanoma model, this strategy significantly increased T-cell infiltration in the tumor tissue.^202^ CAFs also secrete IL-6, which activates the STAT3 signaling pathway to promote tumor cell growth and treatment resistance.^203^ To counteract this, siRNA liposome nanoparticles can silence IL-6 expression in CAFs, block inflammatory signals, reduce tumor resistance, and improve chemotherapy sensitivity.^168^ Additionally, fibroblast-specific protein-1 (FSP-1), which is highly expressed in CAFs, directly contributes to CAF migration and tumor metastasis.^204^ Gene inhibitor nanoparticles can significantly reduce CAF migration, thereby decreasing the formation of tumor metastases. CAF-secreted extracellular vesicles carrying miRNAs, such as miR-21 and miR-92a, regulate tumor cell growth and metastasis.^186^ Nanoparticles mimicking extracellular vesicles can block the signaling of CAF-secreted exosomes by delivering anti-miRNA drugs, such as miR-92a inhibitors. This method significantly reduces metastatic focus formation and improves chemotherapy effectiveness in colorectal cancer.^205^ Finally, CAFs regulate their own activation and the immunosuppressive tumor microenvironment through the transforming growth TGF-β signaling pathway.^206^ Nanoparticles carrying TGF-β inhibitors can significantly reduce tumor fibrosis, and immune nanoparticles that inhibit both TGF-β and PD-L1 signaling secreted by CAFs improve the effectiveness of immunotherapy.^207^

The biomarkers expressed by CAFs provide many potential targets for tumor treatment, and nanomaterials targeting these markers can improve treatment accuracy and effectiveness. By using methods like gene silencing, blocking receptors, and regulating the immune system, nanomaterials can control how CAFs affect the tumor microenvironment, addressing the limits of traditional treatments. In the future, combining nanotechnology with precision medicine is expected to make CAF-targeted nanomaterials more important for personalized cancer therapy.

### E. Nanomaterials targeting TME and CAFs

**FIG. 3.**
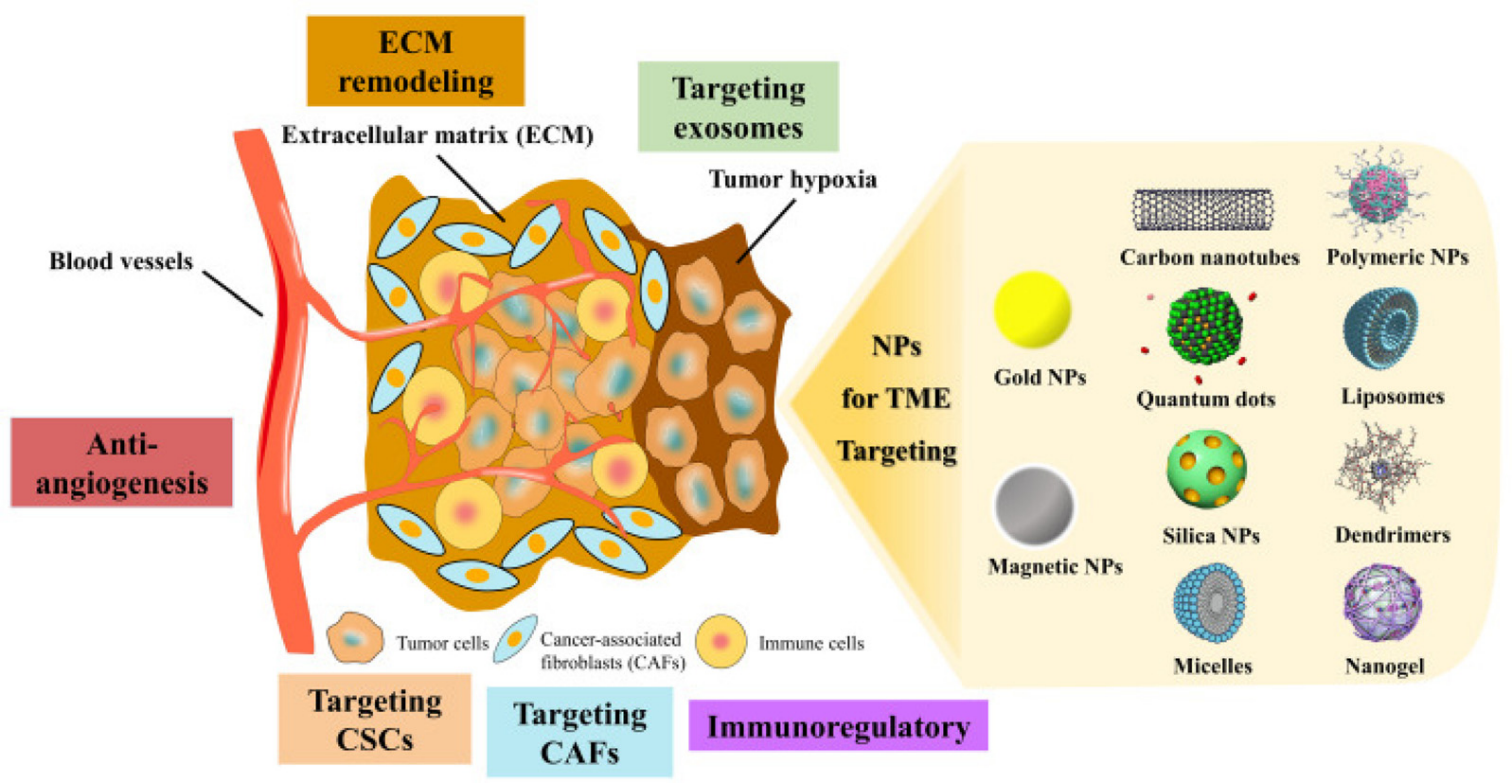
Schematic representation of the TME and nanoparticle-based therapeutic strategies. The TME is composed of tumor cells, CAFs, immune cells, and ECM, which collectively contribute to tumor progression and therapy resistance. Major strategies for targeting the TME include ECM remodeling, targeting exosomes, alleviating tumor hypoxia, anti-angiogenesis, targeting cancer stem cells (CSCs), targeting CAFs, and immunomodulation. Various nanoparticle platforms, including gold nanoparticles, quantum dots, carbon nanotubes, polymeric nanoparticles, liposomes, silica nanoparticles, dendrimers, micelles, magnetic nanoparticles, and nanogels, are employed to enhance therapeutic delivery and efficacy within the TME. These approaches aim to overcome the inherent complexity of the TME and improve treatment outcomes. Reproduced with permission from Tang *et al.*, Int. J. Nanomed. **16**, 5811–5829 (2021). Copyright 2021 by the authors.^208^

The functional diversity and high heterogeneity of CAFs make them a complex target for tumor therapy but also provide several potential pathways for precision therapy (Fig. 3). So far, the rapid development of nanotechnology has enabled the use of nanomaterials for therapeutic methods. By targeting CAFs and their interactions with other components in the TME, these nanomaterials can effectively regulate tumor progression driven by CAFs. Despite many studies showing the potential of nanomaterials in CAF-targeted therapy, the dynamic plasticity of CAFs, complex signaling pathways, and the high heterogeneity of the TME remain challenges that current technology cannot fully overcome.^27^ Therefore, exploring how nanomaterials can target key components of CAFs and the TME, and how to achieve more precise tumor treatment based on this, is of great research value.

First, CAFs promote excessive deposition of ECM to harden the tumor stroma, thereby limiting effective drug delivery.^209^ To address this, collagenase-loaded nanoparticles can break down collagen secreted by CAFs, significantly improving drug penetration.^183^ In addition, matrix metalloproteinase (MMP)-responsive nanomaterials can accurately release drugs to the tumor site through their specific response to CAF-secreted enzymes.^210^ Meanwhile, in photothermal therapy, gold nanoparticles (AuNPs) not only disrupt the integrity of ECM by heating local areas but also enhance the infiltration of immune cells.^211^ Although these strategies have shown significant effects in preclinical models, excessive ECM degradation can cause tumor cells to shed and metastasize, so the level of degradation must be carefully controlled in application.^212^

At the same time, unlike directly clearing CAFs, reprogramming their pro-tumor phenotype to restore their normal function has been a highly anticipated treatment strategy in recent years. For example, DNA origami nanostructures are used to deliver epigenetic regulators, thereby reprogramming CAFs from a pro-tumor phenotype to a quiescent or anti-tumor phenotype.^213,214^ In addition, dual-drug-loaded nanoparticles can simultaneously deliver retinoic acid and TGF-β inhibitors, achieving dual effects of fibrosis inhibition and CAF function normalization.^215^ In preclinical studies, these strategies significantly reduced fibrosis and enhanced the efficacy of immunotherapy. However, current research on the molecular characteristics of CAF subtypes is not sufficient to effectively distinguish between pro-tumor and anti-tumor CAF subgroups, which poses a challenge for precise reprogramming therapy.^216^

Furthermore, CAFs construct a microenvironment for tumor immune escape by expressing PD-L1 and recruiting immunosuppressive cells such as regulatory T cells (Tregs).^217^ In response, immune checkpoint inhibitor nanoparticles can significantly restore T cell function and improve the effectiveness of immunotherapy by targeting the PD-L1 expression induced by CAFs.^218^ In addition, nanoparticles simulating exosomes not only activate anti-tumor immune responses by delivering tumor antigens and adjuvants but also reshape the immune balance of TME to a certain extent.^219,220^ More innovatively, nanoparticles integrated with biosensors can monitor immune suppression signals induced by CAFs in real time and release corresponding therapeutic drugs based on the results.^221^ However, the immune suppression mechanism driven by CAFs is complex, and relying on a single immune regulation strategy may not fully reverse this process. Thus, combining other therapeutic approaches may be necessary.

With the continuous development of nanotechnology, combining artificial intelligence (AI) and biosensors has further enhanced the multifunctionality of CAF-targeted therapy. AI-driven nanomaterial design can improve CAF targeting efficiency by optimizing material size, shape, and surface properties.^222^ For instance, AI can predict the various effects of nanomaterials on CAF signaling pathways, allowing for the design of therapies that target multiple pathways simultaneously.^223,224^ In addition, nanoparticles integrated with biosensors provide the possibility of real-time monitoring of TME changes, making treatment more precise.^225^ However, despite the promising potential of AI and sensor technologies, their application in clinical settings still faces challenges, including technical complexity and regulatory barriers. While CAF-targeting nanomaterials have made significant strides in tumor therapy, they still face several limitations. For example, the heterogeneity and functional plasticity of CAFs increase the complexity of targeted therapy, and off-target effects of nanomaterials remain a key safety concern.^226^ Moreover, large-scale production of nanomaterials faces challenges related to high costs and quality control. The future direction should involve combining single-cell omics technology to deeply analyze the subtype characteristics of CAFs and developing more precise, personalized treatment plans.^227,228^ At the same time, optimizing nanomaterial surface modification technologies can further reduce toxicity and improve specificity.^229^ Additionally, combining CAF-targeted therapy with traditional treatments (such as chemotherapy and radiotherapy) or immunotherapy could significantly enhance overall treatment effectiveness.^108^

Nanomaterials offer various therapeutic strategies for targeting tumor-associated fibroblasts and controlling the tumor microenvironment. From ECM breakdown to immune regulation and phenotype changes, these approaches show the wide potential of nanotechnology in cancer treatment. The collaboration between AI, biosensors, and advanced biotechnology will further help the use of CAF-targeted nanomaterials in personalized cancer treatment.

### F. Quantitative analysis of *in vivo*/*ex vivo* accumulation of nanoparticles in tumors

**FIG. 4.**
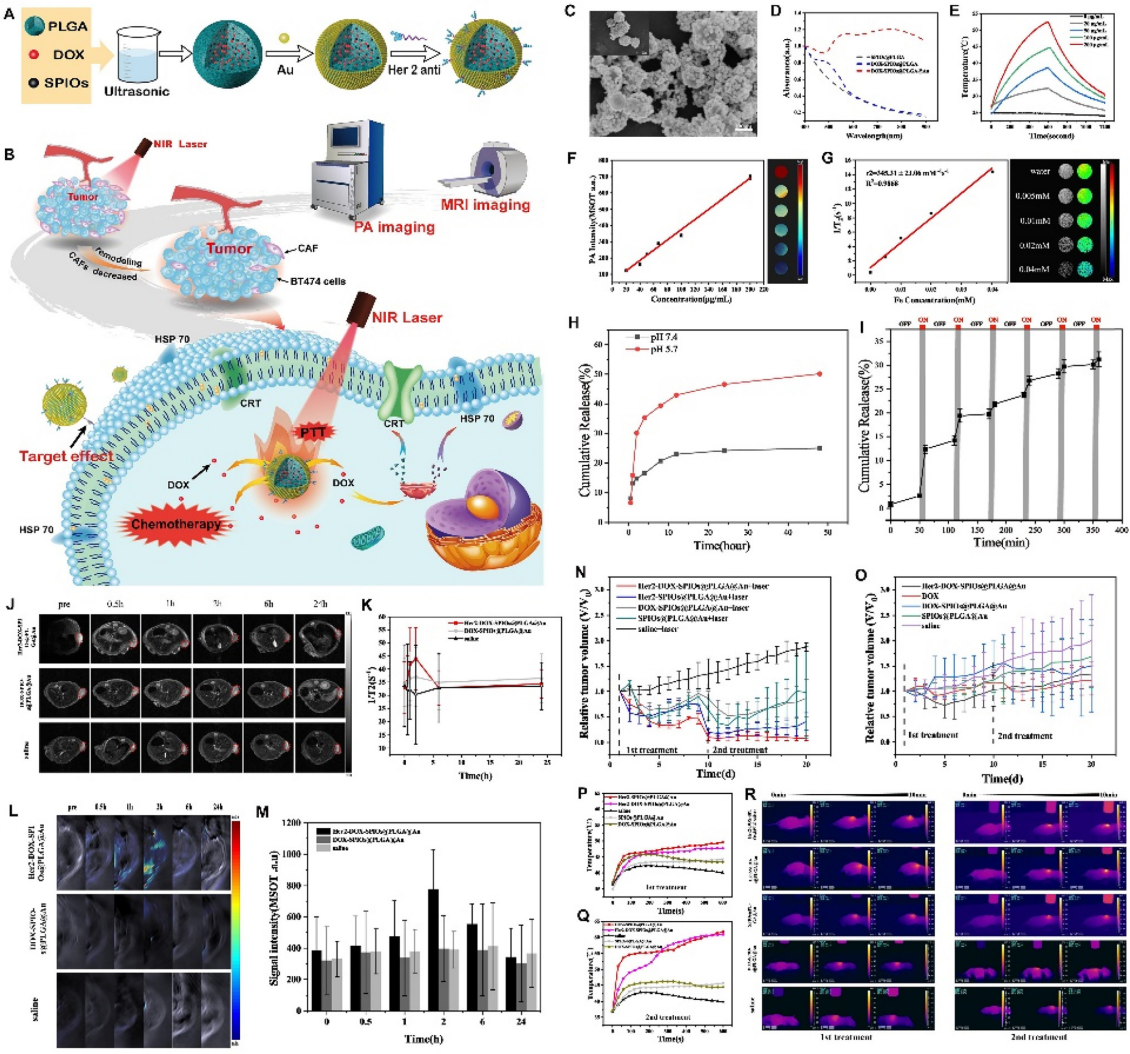
(a) and (b) Schematic illustration of the structure and application of Her2-DOX-SPIOs@PLGA@Au NPs. (a) The composition and fabrication process of Her2-DOX-SPIOs@PLGA@Au NPs. (b) The nanoscale theranostic agent is utilized for PA/MRI dual-modal imaging and tumor elimination through chemical-photothermal therapy and immune activation. (c-i) These figures demonstrate the characterization and performance of DSG NPs, including their imaging capabilities, temperature response under NIR laser irradiation, drug release behavior under different conditions, and magnetic resonance properties. (j)–(m) These figures demonstrate the *in vivo* imaging of a BT474 mice xenograft tumor to evaluate the accumulation and distribution of nanoparticles, particularly in CAFs-rich regions, which is crucial for assessing their therapeutic efficacy and specificity. (j) T2-weighted MR imaging of the tumor at different time points (0, 0.5, 1, 2, 6, and 24 h), highlighting the tumor (red dashed circles) to monitor nanoparticle distribution over time. (k) T2 relaxation rate [1/T2(s−1)] of the tumor's region of interest (ROI) at different time points, showing how the particles interact with the tumor environment. (l) Photoacoustic imaging at the same time points, capturing the contrast of nanoparticles within the tumor for better understanding of their location and accumulation. (m) Signal intensity analysis from the ROI of the tumor over time, providing quantitative data on nanoparticle distribution and accumulation. Data are expressed as mean ± SD, n = 3. (n)–(r) These figure illustrate the effects of various treatments on tumor growth and temperature variations during the monitoring period. These results demonstrate the impact of different nanoparticle treatments and laser irradiation on tumor growth inhibition and temperature changes, providing insight into their potential for enhanced cancer therapy. (n) and (o) The curve of relative tumor volume variations with different treatments during the 20-day monitoring period, the first treatment and second treatment were performed on day 1 and day 10, respectively, data expressed as mean ± SD, n = 3. (p) and (q) The temperature variations of tumor under laser irradiation (10 min, 808 nm, 1 W/cm^2^) during first treatment and second treatment. (r) IR thermal images of various treatment groups. Reproduced with permission from Zheng *et al.*, Int. J. Nanomed. **15**, 10007–10028 (2020). Copyright 2020 by the authors.^230^

Quantitative analysis of nanoparticle accumulation in tumors, especially in CAFs-rich areas, is important for assessing their therapeutic effectiveness and specificity. This analysis helps understand how nanoparticles distribute in the body and how they move through the system. It also explains the complex relationship between nanoparticles and the TME (Fig. 4). A thorough evaluation of nanoparticle accumulation in tumors, both in living organisms and in laboratory settings, is necessary to improve their design and use in clinical practice. However, the lack of consistent quantitative data across studies limits the ability to apply current findings to real-world situations. This calls for a more systematic and thorough approach.

Quantitative assessment serves several important purpose, such as measuring how specifically nanoparticles target CAFs-rich areas, evaluating their ability to pass through dense ECM barriers, and understanding their clearance and retention patterns. Measures like tumor-to-normal tissue ratio (TNR), percentage injected dose per gram of tissue (%ID/g), and retention time allow researchers to track nanoparticle localization and treatment effects.^231–233^ These analyses are also crucial for determining how effectively nanoparticles influence CAF-driven processes, like ECM remodeling and immune suppression.^4^ *In vivo* analysis mostly uses imaging and other techniques to track nanoparticle distribution in living organisms. For example, fluorescently labeled nanoparticles allow real-time tracking of tumor accumulation, while advanced imaging methods like PET and MRI provide high-sensitivity images of radiolabeled or magnetic nanoparticles.^234,235^ Additionally, mass spectrometry techniques, such as ICP-MS, give precise measurements of nanoparticle content in tumor tissues after sacrifice.^236^ Therefore, metrics like %ID/g or TNR provide valuable information on the specificity and effectiveness of nanoparticle delivery. However, these results are often limited by the changing nature of the TME, which can alter how nanoparticles are distributed over time.^237^ Thus, a combination of *in vivo* imaging and postmortem analysis is often needed for a more complete understanding.

To complement *in vivo* studies, *ex vivo* analysis provides clearer data on nanoparticle distribution within tumor tissues. Techniques like confocal microscopy and histopathology help visualize nanoparticle localization in relation to CAF markers such as FAP or α-SMA.^238^ Furthermore, flow cytometry can measure nanoparticle uptake by specific cell types, including CAFs, tumor cells, or immune cells.^239^ Combining *ex vivo* results with *in vivo* imaging is important for a better understanding of nanoparticle behavior. Additionally, *ex vivo* analysis helps assess penetration depth, a key factor in ECM-dense tumors like PDAC.^240^ For instance, collagenase-loaded nanoparticles increased tumor penetration by 1.8 times compared to conventional systems, highlighting the importance of ECM-targeting strategies.^183^

Quantitative analysis of nanoparticle accumulation faces several challenges. First, tumor heterogeneity, including variations in CAF density, ECM composition, and vascularization, makes it difficult to compare results between studies. Second, dynamic interactions between nanoparticles and the TME, like opsonization and clearance, often reduce targeting efficiency. Third, the lack of standardized methods for measuring nanoparticle accumulation complicates comparisons across studies. Finally, in tumors with dense ECM, poor nanoparticle penetration into deeper areas limits therapeutic effectiveness. Addressing these challenges is crucial for advancing nanoparticle-based therapies in clinical settings.

## IV. EMERGING NANOMEDICINE APPROACHES FOR PRECISION TARGETING OF CAFs

CAFs play a pivotal role in tumor progression, metastasis, and resistance to treatment, making them an attractive target for precision medicine. Nanomedicine, which utilizes nanoscale drug delivery systems, has emerged as a powerful approach to specifically target CAFs, improving therapeutic outcomes while minimizing off-target effects.^26,241,242^ This section discusses innovative nanomedicine strategies designed to target CAFs, focusing on novel nanoscale drug delivery platforms, surface functionalization techniques, environmentally responsive nanocarriers, and multi-drug nanocarriers for combination therapy. By exploring these approaches, we aim to highlight the potential of nanomedicine in addressing the complex tumor microenvironment and enhancing the effectiveness of cancer treatments.

### A. Innovative nanoscale drug delivery systems for targeting CAFs

**FIG. 5.**
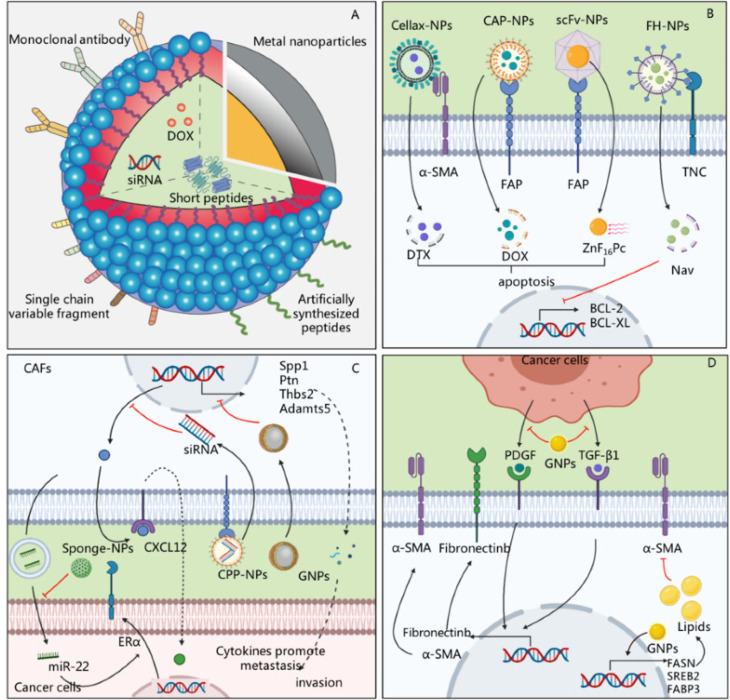
Innovative nanoscale drug delivery systems for targeting CAFs in the tumor microenvironment. (a) Multifunctional nanoparticles, incorporating monoclonal antibodies, short peptides, siRNA, doxorubicin (DOX), and metal nanoparticles, are engineered for targeted delivery to CAFs. (b) Advanced nanoparticle systems such as Cellax-NPs, CAP-NPs, scFv-NPs, and FH-NPs target specific CAF markers, including α-SMA, FAP, and TNC, to induce apoptosis and inhibit survival signaling pathways like BCL-2 and BCL-XL. (c) siRNA-loaded nanoparticles and sponge-like NPs modulate CAF-driven processes, such as CXCL12 secretion and cytokine production, which promote tumor invasion and metastasis. They also target specific pathways, such as ERα regulation by miR-22, to reduce CAF activity. (d) Gold nanoparticles (GNPs) and other delivery platforms disrupt CAF-mediated fibrosis and lipid metabolism by targeting pathways involving PDGF, TGF-β1, α-SMA, fibronectin, and lipid metabolism regulators (e.g., FASN, SREBP2, FABP3). Reproduced with permission from Mu *et al.*, Int. J. Mol. Sci. **22**(21), 11671 (2021). Copyright 2021 by the authors.^112^

Nanoscale drug delivery systems have become a critical focus in cancer treatment due to their ability to precisely target CAFs. CAFs play an essential role in tumor growth, metastasis, and chemotherapy resistance, making them an attractive therapeutic target (Fig. 5). Therefore, developing nanosystems that can efficiently deliver drugs to CAFs while minimizing off-target effects is of paramount importance.

Several new nanoscale drug delivery platforms have been developed, showing unique properties that make them well-suited for targeting CAFs. First, ultrafine nanoparticles and nanomicelles, with their small size and large surface area, have shown great effectiveness in tumor treatment.^20,243^ These nanoparticles, typically ranging from 1 to 100 nm, are small enough to take advantage of the enhanced EPR effect, allowing them to accumulate in tumor tissue.^244^ Additionally, nanomicelles, due to their ability to encapsulate hydrophilic drugs, offer enhanced stability and controlled release, which improves drug bioavailability.^245^ By modifying the surfaces of these nanoparticles or micelles with specific ligands, such as antibodies or targeting peptides for CAFs, their ability to selectively target CAFs can be greatly improved, enhancing therapeutic efficacy.^246,247^

Liposomes, one of the most established types of nanocarriers, have been widely studied for their biocompatibility and controlled drug release properties.^248^ For example, the liposomal drug Doxil (liposomal doxorubicin) has been successfully used in clinical settings.^249,250^ Its lipid bilayer structure protects the drug from premature degradation and ensures sustained release within the tumor.^251^ By modifying liposomes with polyethylene glycol (PEG), their circulation time in the bloodstream is extended, which reduces clearance by the immune system.^252^ When further modified with antibodies or specific peptides targeting CAFs, liposomes can enhance their ability to target CAFs, improving therapeutic outcomes.^253^ Carbon nanotubes (CNTs) are a new class of nanocarriers with great potential due to their unique structure. With a high surface area and excellent drug-loading capacity, CNTs can carry a large amount of drug and deliver it to the tumor site through the bloodstream.^254,255^ The surface of CNTs can be modified with specific targeting peptides or antibodies, allowing for selective binding to CAFs.^256^ Additionally, CNTs can respond to changes in the tumor microenvironment, such as acidic conditions or high hydrogen peroxide levels, to release drugs in a controlled way.^257^ As such, CNTs show great promise as a delivery platform for targeting CAFs in future applications. However, designing the ideal nanocarrier is still challenging. In addition to drug loading capacity, the size, shape, and surface charge of nanoparticles greatly affect their distribution and targeting efficiency.^258^ Smaller nanoparticles (typically 10–100 nm) are more likely to penetrate tumor blood vessels and accumulate within the tumor.^259^ The shape of the nanoparticle also affects how it interacts with the cell membrane. For instance, spherical nanoparticles generally have better stability and higher cellular uptake compared to non-spherical shapes.^260^ By carefully controlling the size and shape of nanoparticles, both their targeting efficiency and ability to penetrate the tumor can be optimized.

The surface charge of nanoparticles is another important factor that affects their behavior *in vivo*. Generally, negatively charged nanoparticles show better biocompatibility and lower immunogenicity, while positively charged nanoparticles tend to adsorb more strongly to cell membranes, increasing cellular uptake.^261,262^ Therefore, designing the surface charge carefully can improve the targeting ability and drug delivery efficiency of nanocarriers.

### B. Surface functionalization strategies for targeting CAFs

Surface functionalization is a critical aspect of enhancing the specificity and efficacy of nanoscale drug delivery systems for targeting CAFs. To achieve precise targeting, it is essential to identify the appropriate biomarkers expressed on CAFs.^144^ Among the most studied markers are fibronectin and α-SMA. Fibronectin is an extracellular matrix protein overexpressed in the tumor stroma, while α-SMA is a marker linked to activated fibroblasts.^263,264^ These markers are ideal candidates for nanoparticle functionalization because they are highly expressed in the tumor microenvironment but found minimally in normal tissues.^265^ Additionally, integrins like α_v_β_3_ and α_v_β_5_ are commonly expressed on CAFs and play a role in cell adhesion to the extracellular matrix.^143^ These integrins are also good targets for nanoparticles, providing a solid foundation for the development of surface-modified nanocarriers.^143,266^ Therefore, selecting the right targeting markers is crucial for the success of functionalized drug delivery systems aimed at CAFs.

Another promising functionalization strategy uses carbohydrates, such as mannose and galactose, to target CAFs.^144,267^ These carbohydrates can be attached to nanoparticles to interact with cell surface lectins or carbohydrate-binding receptors that are overexpressed on CAFs.^268^ While this strategy provides an alternative to antibody- and peptide-based functionalization, it generally offers lower binding affinity and specificity. However, carbohydrates are relatively easy to modify and can be combined with other ligands to improve targeting efficiency.^194,269^ This approach is especially useful for targeting CAFs with lectin receptors, which are present in the tumor microenvironment. The success of surface functionalization strategies depends largely on the receptor–ligand interactions between nanoparticles and CAFs.^144^ For example, the binding between RGD peptides and integrins, or antibodies and CAF-specific antigens like fibronectin or α-SMA, is key to ensuring the specificity and efficiency of drug delivery.^270,271^ The strong affinity of RGD peptides for integrins has been well-documented in many studies, making them a common choice for targeting CAFs.^272^ Similarly, antibody-antigen interactions are highly specific, offering an effective way to guide nanoparticles to CAFs.

Additionally, it is important to recognize the heterogeneity of the tumor microenvironment. CAFs can express different receptor profiles depending on their activation state or the type of tumor, meaning receptor–ligand interactions must be tailored to the specific CAF subtype.^273^ As a result, combining different functionalization approaches, such as dual-targeting strategies, could significantly improve targeting accuracy. Dual-targeting, a promising approach in nanomedicine, involves functionalizing nanoparticles with two distinct ligands: one targeting CAFs and the other targeting tumor cells.^274,275^ This strategy can significantly improve therapeutic efficacy by addressing the complexity of the tumor microenvironment. For example, nanoparticles could be functionalized with RGD peptides to target CAFs and folate to target tumor cells. This combined targeting ensures both the stromal and cancerous components of the tumor are addressed, improving overall therapeutic outcomes.^276,277^ Moreover, dual-targeting strategies could help overcome resistance mechanisms, such as CAF-induced drug resistance, which is common in single-target therapies.

### C. Environmentally responsive nanocarriers for CAFs-targeted drug delivery

The unique characteristics of the TME offer many opportunities for developing responsive nanocarriers in cancer treatment. The TME is characterized by features like low pH, increased hydrogen peroxide (H_2_O_2_) concentration, and the overexpression of specific enzymes, which can be used as triggering mechanisms for targeted drug delivery systems.^278,279^ Designing nanocarriers that respond to these microenvironmental signals enhances drug targeting and ensures precise drug release at tumor and CAF sites, thereby maximizing therapeutic efficacy.^280–282^

There are significant differences between the TME and normal tissue, with key features including low pH, increased hydrogen peroxide concentration, and the secretion of specific enzymes, such as MMPs by CAFs.^283^ These distinct characteristics provide the basis for designing environment-responsive nanocarriers. Tumor tissue typically has an acidic microenvironment due to active metabolic processes in tumor cells, leading to a decrease in local pH, typically ranging from 6.0 to 6.5—lower than the normal tissue pH of around 7.4.^284,285^ Additionally, hydrogen peroxide concentration in tumor tissues is elevated, and MMP activity is significantly increased due to the influence of CAFs.

The acidic pH commonly found in the TME is a well-known trigger for the development of pH-responsive nanocarriers. These carriers are usually made from materials that degrade or change structure under acidic conditions, such as poly(lactic-co-glycolic acid) copolymers (PLGA), polyethylene imine (PEI), and polystyrene sulfonate (PSS).^286–288^ These materials remain stable at normal tissue pH (∼7.4) but degrade or undergo structural changes when exposed to the lower pH typical of tumor environments, releasing the drug. For example, PLGA is a widely used pH-sensitive material that easily hydrolyzes in acidic environments, releasing the encapsulated drug.^288^ The degradation rate of PLGA is closely linked to the surrounding pH, allowing for rapid breakdown in low pH tumor microenvironments and precise drug delivery near CAFs.^289^ Additionally, some pH-responsive carriers are designed to change their surface charge or hydrophilicity in acidic conditions, facilitating drug release and promoting interaction with the cell membrane.^290,291^ This feature allows pH-responsive nanocarriers to take advantage of the tumor's acidic environment for targeted drug release, improving drug bioavailability and therapeutic efficacy.

Beyond pH response, enzyme-sensitive nanocarriers are crucial for targeting the TME, particularly near CAFs. CAFs secrete MMPs, which are key players in tumor stroma remodeling. MMPs degrade the ECM, aiding tumor cell migration and invasion.^5,210^ As a result, enzyme-responsive nanocarriers have been developed to undergo structural changes or degradation when exposed to MMPs, releasing their drug payloads.^292^ For example, some nanocarriers are functionalized with enzyme-cleavable peptide chains that MMPs can specifically recognize and cleave.^293^ Once the nanocarrier reaches the CAF-rich tumor region and encounters MMPs, the peptide chain is hydrolyzed, causing the nanocarrier to rupture and release the drug. Unlike pH-responsive carriers, enzyme-responsive systems have the advantage of targeting the unique enzyme activity of CAFs, offering higher specificity and better control over drug release.^151^ In addition to MMPs, other enzymes, such as peroxidases and cholesterol esterases, are present in the tumor microenvironment and can also function as response mechanisms.^294,295^ Therefore, designing nanocarriers responsive to multiple enzyme types can improve the precision of drug release and treatment.

Another notable feature of the TME is the elevated concentration of H_2_O_2_, a by-product of the high metabolic activity and redox processes within tumor cells.^296^ This increased concentration of H_2_O_2_ provides an opportunity to develop redox-responsive nanocarriers that release drugs specifically in the tumor region.^297,298^ These carriers are typically made from materials that react with hydrogen peroxide, such as polymers containing thiol groups (-SH) or disulfide bonds (S-S).^298^ When exposed to high levels of H_2_O_2_, these chemical groups undergo a reduction reaction, causing the nanocarrier to break down and release its drug payload.^299^ For example, polymers crosslinked with disulfide bonds can undergo a reduction reaction in the presence of hydrogen peroxide, breaking the carrier into smaller molecules and releasing the drug.^300^ The advantage of redox-responsive carriers is that hydrogen peroxide concentration is usually elevated within the tumor microenvironment, ensuring that drug release is localized to the tumor site and improving therapeutic efficacy.

### D. Multi-drug nanocarriers for CAFs-targeted combination therapy

Nowadays, there is growing interest in combination therapy for cancer treatment, which highlights the need for new drug delivery systems that can effectively deliver multiple therapies simultaneously. Single-drug treatments, such as chemotherapy and immunotherapy, often face limitations like drug resistance and insufficient effectiveness. Therefore, multi-drug nanocarriers have become a promising solution to improve cancer treatment outcomes, especially by targeting both CAFs and tumor cells together. The design of multi-drug nanocarriers aims to deliver multiple therapeutic agents in a single nanoplatform, ensuring controlled and targeted release within the tumor microenvironment.

Multi-drug delivery can be achieved through various physical and chemical methods, including co-loading strategies, composite carrier systems, and targeted modification.^301,302^ In the co-loading strategy, multiple drugs are encapsulated within a single nanocarrier using either covalent bonding or non-covalent interactions.^303^ These drugs can coexist in different regions of the carrier without interfering with each other, maintaining their stability and therapeutic properties.^304^ For example, drugs can be compartmentalized in phase-separated or core-shell structures. This design allows for the independent release of each drug when triggered by specific environmental conditions, improving the therapeutic outcome.^305^ The co-loading strategy is especially beneficial when combining drugs with complementary mechanisms of action, such as chemotherapeutic agents and immunotherapies, to achieve synergistic effects.

In composite carrier design, different materials are combined to create a nanocarrier that can load multiple drugs at once. For example, liposomes, polymeric nanoparticles, and metallic nanostructures can be integrated to improve drug loading capacity, stability, and controlled release.^306^ A particularly effective example is the combination of polymeric nanoparticles and liposomes, which increases the payload capacity while stabilizing the loaded drugs.^307^ Additionally, composite carriers ensure that drugs are released in a controlled manner based on the specific needs of the tumor microenvironment, maximizing therapeutic efficacy and minimizing systemic side effects.^308^

The surface targeting modification strategy aims to improve drug delivery precision by functionalizing the surface of nanocarriers with ligands that specifically recognize CAFs and tumor cells. This is achieved by attaching antibodies, peptides, or other targeting molecules to the carrier surface, allowing them to bind to specific receptors or antigens on the tumor or CAF surface. This functionalization not only enhances the delivery of nanocarriers to the tumor site but also adds an extra layer of specificity by targeting the unique components of the tumor microenvironment.^248^ By targeting both CAFs and tumor cells, this approach ensures that therapeutic agents are delivered to the right areas, improving treatment outcomes.^111^ One key advantage of combination therapy is its ability to overcome the limitations of single-agent treatments and improve therapeutic efficacy through synergistic effects.^309^ For instance, a typical combination therapy involves chemotherapy drugs, such as docetaxel, and immune checkpoint inhibitors, such as PD-1 inhibitors, which work together.^310^ By loading both chemotherapy and immune drugs onto nanocarriers and modifying them to target CAFs and tumor cells, precise drug release can be achieved at the tumor site.^23,311^ Targeting molecules on the nanocarriers bind to specific receptors on CAFs first, reducing the impact of the drugs on normal tissues.^312^ Meanwhile, another portion of the drugs is released near tumor cells, enhancing the overall effectiveness of combination therapy.

The combination therapy strategy using multi-drug nanocarriers shows great potential for improving treatment precision and efficacy by simultaneously targeting CAFs and tumor cells. Through the synergistic effects of chemotherapy, targeted therapies, and immune drugs, combination therapy can overcome drug resistance and reduce toxic side effects on healthy tissues by optimizing drug distribution within the tumor microenvironment.^313,314^ With ongoing advancements in nanotechnology, future multi-drug nanocarriers are likely to integrate even more targeting mechanisms and release regulation strategies, further improving the overall effectiveness of cancer treatment.

### E. Preclinical advancements in nanomedicines targeting CAFs: Drug delivery strategies and clinical application challenges

In the preclinical stage, targeted therapies for CAFs have made significant strides across various animal models (Table III). Studies have demonstrated that nanodrug delivery systems, such as liposomes and polymer nanoparticles, along with surface modification strategies like antibodies and peptides, can effectively enhance the selective targeting of drugs to CAFs.^111,269,312^ These strategies also improve drug accumulation in tumor tissues, contributing to more efficient treatments. Additionally, combination therapy approaches, such as the use of chemotherapy drugs in conjunction with immunotherapy, have shown promising results in preclinical studies. By leveraging the synergistic effects of multiple drugs, these strategies not only enhance tumor therapeutic outcomes but also help overcome resistance that may arise from relying on a single drug.

**TABLE III.** Current preclinical and clinical application progress of nanomedicines targeting CAFs.

Nanocarrier type	Drug types	Preclinical research progress	Clinical application potential and challenges	Refs.
Liposomes	Chemotherapy drugs (e.g., Doxorubicin), immune checkpoint inhibitors (e.g., PD-1 inhibitors)	1. Enhanced targeting of CAFs, increased drug accumulation in the tumor microenvironment. 2. Good selectivity and biocompatibility in animal models.	1. Improving penetration into the tumor microenvironment. 2. Stability and scalability challenges in clinical translation. 3. FDA approval, manufacturing, and quality control issues.	315–317
Polymeric nanoparticles	Immune drugs (e.g., Anti-PD-1 antibody), chemotherapy drugs (e.g., Docetaxel)	1. Effectively co-loads multiple drugs, improving dual targeting to CAFs. 2. Significant antitumor effects in animal models.	1. Addressing drug loading efficiency and nanoparticle stability. 2. Challenges in biodegradability and clearance rate optimization for clinical application.	318,319
Gold nanoparticles	Chemotherapy drugs, targeted drugs (e.g., antibody targeting CAFs or tumor vasculature)	1. Good biocompatibility and strong photothermal therapy effects. 2. Enhanced drug accumulation in tumors through surface modifications.	1. Production scalability and cost control. 2. Further validation of biocompatibility and immunogenicity in humans.	320,321
Nanomicelles	Chemotherapy drugs (e.g., Paclitaxel), targeted drug conjugates (e.g., ADCs)	1. Surface modification (e.g., PEGylation) increases drug half-life and targeting ability. 2. Effective in reducing systemic toxicity in animal studies.	1. Drug loading and release control still represent a bottleneck for clinical use. 2. Stability and long-term stability issues remain in clinical settings.	322,323
Nanoemulsions	Chemotherapy drugs, immune drugs, targeted small molecule drugs	1. Significantly increases drug concentration in tumors and improves CAF targeting. 2. Shows good antitumor activity and low toxicity in animal models.	1. Stability and storage challenges for clinical applications. 2. Need further investigation into human safety and efficacy.	324,325
Nanotubes	Chemotherapy drugs (e.g., Cyclophosphamide), targeted drugs (e.g., antibodies, peptides)	1. High surface area and loading capacity, suitable for co-delivery of multiple drugs. 2. Effective targeting of CAFs and enhanced antitumor effects in animal studies.	1. Clinical challenges include optimizing drug loading, release rates, and biocompatibility. 2. Issues with controlled drug release and long-term stability.	254,326

Although nanocarrier delivery systems targeting CAFs have shown promising results in preclinical studies, their clinical translation still faces significant challenges. Future research should focus on optimizing nanocarrier design, improving drug targeting and penetration within the tumor microenvironment, addressing stability and pharmacokinetic concerns, and overcoming production and regulatory hurdles.^26,111,144^ As research deepens and more clinical data are accumulated, nano drug delivery systems targeting CAFs are expected to become a key component of personalized cancer treatment, offering more effective and less toxic therapeutic strategies.

## V. POTENTIAL STRATEGIES AND FUTURE DIRECTIONS FOR TARGETING CAFs

CAFs play diverse and complex roles in the TME, and their potential for targeted therapy has gained widespread recognition.^39,327^ Beyond the application of nanomaterials, recent advancements in technologies such as biomedical engineering, biomaterials, tissue engineering, microfluidics, synthetic biology, and medical devices have further expanded the possibilities for CAFs targeting.^112,328^ In the realm of biomedical engineering and biomaterials, functional hydrogels, stimulus-responsive polymers, and exosome-mimicking materials not only facilitate accurate drug delivery but also effectively regulate the remodeling of the CAFs-driven ECM, overcoming drug penetration barriers.^329–331^ These advanced biomaterials, thus, provide a technological foundation for the targeted therapy of CAFs. Additionally, tissue engineering techniques, such as three-dimensional (3D) bioprinting models and tumor-on-a-chip platforms, enable the simulation of CAF dynamics within the TME.^332–334^ These innovations enhance drug screening efficiency and provide a deeper understanding of the interactions between CAFs and other components of the microenvironment, offering valuable experimental data for developing novel therapies.^335^ Meanwhile, microfluidic technology significantly enhances the specificity of targeted therapy through high-precision single-cell separation and drug delivery.^336,337^ Notably, the development of tumor microenvironment co-culture systems allows for real-time monitoring of CAF functions, providing crucial data for optimizing treatment combinations.^338^ Synthetic biology further broadens the scope of intelligent therapies. By using tools like CRISPR-Cas9 and synthetic gene circuits, researchers can target and knock out tumor-promoting genes in CAFs or dynamically regulate their phenotype, transforming them into an anti-tumor state.^339,340^ This enables multidimensional intervention in CAFs.

In parallel, medical devices such as implantable biosensors and catheter-based drug delivery systems offer innovative solutions for CAFs monitoring and intervention, particularly in precision treatment and real-time dynamic adjustments.^341,342^ These devices show enormous potential for clinical application. The synergistic integration of these technologies not only compensates for the limitations of individual treatment strategies but also opens up broader research possibilities for CAFs-targeted therapy. However, these technologies also face challenges. The heterogeneity and dynamic plasticity of CAFs increase the complexity of targeted therapy, while the clinical translation of these advanced technologies encounters high costs and regulatory hurdles.^31,343^ To overcome these challenges, future research should leverage technologies like single-cell genomics and proteomics to deeply analyze CAF subtype characteristics and optimize the production processes of biomaterials through interdisciplinary collaboration.^37,344,345^ In conclusion, with the further integration of biomedical engineering, tissue engineering, microfluidics, synthetic biology, and medical devices with nanomaterials, CAFs-targeted therapy is poised to play a pivotal role in precision medicine and personalized cancer treatment, offering a comprehensive approach to improving patient outcomes.

## Data Availability

Data sharing is not applicable to this article as no new data were created or analyzed in this study.

## NOMENCLATURE

AI: Artificial intelligence
apCAF: Antigen-presenting cancer-associated fibroblasts
AuNPs: Gold nanoparticles
BCL-2: B-cell lymphoma 2
BCL-XL: B-cell lymphoma-extra large
BSA: Bovine serum albumin
CAFs: Cancer-associated fibroblasts
CD4+ T cells: CD4-positive T cells
CD8+ T cells: CD8-positive T cells
CNTs: Carbon nanotubes
CRISPR-Cas9: Clustered regularly interspaced short palindromic repeats and CRISPR-associated protein 9
CTLA-4: Cytotoxic T-lymphocyte-associated protein 4
CTLs: Cytotoxic T lymphocytes
CXCL12: C-X-C motif chemokine ligand 12
CXCR4: C-X-C chemokine receptor type 4
CYP450: Cytochrome P450
DOX: Doxorubicin
ECM: Extracellular matrix
EGFR: Epidermal growth factor receptor
EPCAM: Epithelial cell adhesion molecule
EPR: Enhanced permeability and retention
Exosomes: Extracellular vesicles
FABP3: Fatty acid-binding protein 3
FAP: Fibroblast activation protein
FAP-targeted NPs: Fibroblast activation protein-targeted nanoparticles
FAP-α: Fibroblast activation protein alpha
FASN: Fatty acid synthase
FDA: Food and Drug Administration
FeNPs: Iron oxide nanoparticles
FRT: Fibroblast-representative tumor
FSP-1: Fibroblast-specific protein-1
HGF: Hepatocyte growth factor
iCAF: Inflammatory cancer-associated fibroblasts
IL-10: Interleukin-10
IL-6: Interleukin-6
IL-6R: Interleukin-6 receptor
iNOS: Inducible nitric oxide synthase
LNPs: Lipid nanoparticles
mAb: Monoclonal antibody
MALDI-TOF: Matrix-assisted laser desorption/ionization time-of-flight
MDSCs: Myeloid-derived suppressor cells
MHC: Major histocompatibility complex
MHCII: Major histocompatibility complex class II
MMP: Matrix metalloproteinase
MMPs: Matrix metalloproteinases
MNPs: Magnetic nanoparticles
MOFs: Metal-organic frameworks
myCAF: Myofibroblastic cancer-associated fibroblasts
NDDS: Nanomedicine delivery systems
NF-*κ*B: Nuclear factor kappa-light-chain-enhancer of activated B cells
NIR: Near-infrared
NK cells: Natural killer cells
NMR: Nuclear magnetic resonance
NMs: Nanomaterials
PD-1: Programmed cell death protein 1
PDGFR: Platelet-derived growth factor receptor
PD-L1: Programmed death-ligand 1
PDT: Photodynamic therapy
PEG: Polyethylene glycol
PGE2: Prostaglandin E2
PLGA: Poly(lactic-co-glycolic acid)
PLGA-NPs: Poly(lactic-co-glycolic acid) nanoparticles
RGD: Arginine-glycine-aspartic acid (peptide)
Rho GTPases: Ras homologous GTPases
RNA: Ribonucleic acid
scFv: Single-chain variable fragment
SDF-1: Stromal cell-derived factor 1
SeNPs: Selenium nanoparticles
siRNA: Small interfering RNA
SOSG: Singlet oxygen sensor green
SREBP2: Sterol regulatory element-binding protein 2
TAMs: Tumor-associated macrophages
TEM: Tumor extracellular matrix
TGF-β: Transforming growth factor-beta
TGF-β1: Transforming growth factor-beta 1
TME: Tumor microenvironment
TNF-α: Tumor necrosis factor-alpha
Tregs: Regulatory T cells
vCAF: Vascular cancer-associated fibroblasts
VEGF: Vascular endothelial growth factor
VEGFR: Vascular endothelial growth factor receptor
α-SMA: α-smooth muscle actin
%ID/g: Percentage injected dose per gram of tissue
